# CT-based body composition analysis and pulmonary fat attenuation volume as biomarkers to predict overall survival in patients with non-specific interstitial pneumonia

**DOI:** 10.1186/s41747-024-00519-0

**Published:** 2024-10-14

**Authors:** Luca Salhöfer, Francesco Bonella, Mathias Meetschen, Lale Umutlu, Michael Forsting, Benedikt M. Schaarschmidt, Marcel Opitz, Nikolas Beck, Sebastian Zensen, René Hosch, Vicky Parmar, Felix Nensa, Johannes Haubold

**Affiliations:** 1 Institute of Diagnostic and Interventional Radiology and Neuroradiology, University Hospital Essen, Essen, Germany; 2 Institute for Artificial Intelligence in Medicine, University Hospital Essen, Essen, Germany; 3 Center for Interstitial and Rare Lung Diseases, Department of Pneumology, University Hospital Essen, Essen, Germany

**Keywords:** Body composition, Deep learning, Lung diseases (interstitial), Survival analysis, Tomography (x-ray computed)

## Abstract

**Background:**

Non-specific interstitial pneumonia (NSIP) is an interstitial lung disease that can result in end-stage fibrosis. We investigated the influence of body composition and pulmonary fat attenuation volume (CTpfav) on overall survival (OS) in NSIP patients.

**Methods:**

In this retrospective single-center study, 71 NSIP patients with a median age of 65 years (interquartile range 21.5), 39 females (55%), who had a computed tomography from August 2009 to February 2018, were included, of whom 38 (54%) died during follow-up. Body composition analysis was performed using an open-source nnU-Net-based framework. Features were combined into: Sarcopenia (muscle/bone); Fat (total adipose tissue/bone); Myosteatosis (inter-/intra-muscular adipose tissue/total adipose tissue); Mediastinal (mediastinal adipose tissue/bone); and Pulmonary fat index (CTpfav/lung volume). Kaplan–Meier analysis with a log-rank test and multivariate Cox regression were used for survival analyses.

**Results:**

Patients with a higher (> median) Sarcopenia and lower (< median) Mediastinal Fat index had a significantly better survival probability (2-year survival rate: 83% *versus* 71% for high *versus* low Sarcopenia index, *p* = 0.023; 83% *versus* 72% for low *versus* high Mediastinal fat index, *p* = 0.006). In univariate analysis, individuals with a higher Pulmonary fat index exhibited significantly worse survival probability (2-year survival rate: 61% *versus* 94% for high *versus* low, *p* = 0.003). Additionally, it was an independent risk predictor for death (hazard ratio 2.37, 95% confidence interval 1.03–5.48, *p* = 0.043).

**Conclusion:**

Fully automated body composition analysis offers interesting perspectives in patients with NSIP. Pulmonary fat index was an independent predictor of OS.

**Relevance statement:**

The Pulmonary fat index is an independent predictor of OS in patients with NSIP and demonstrates the potential of fully automated, deep-learning-driven body composition analysis as a biomarker for prognosis estimation.

**Key Points:**

- This is the first study assessing the potential of CT-based body composition analysis in patients with non-specific interstitial pneumonia (NSIP).
- A single-center analysis of 71 patients with board-certified diagnosis of NSIP is presented
- Indices related to muscle, mediastinal fat, and pulmonary fat attenuation volume were significantly associated with survival at univariate analysis.
- CT pulmonary fat attenuation volume, normalized by lung volume, resulted as an independent predictor for death.

**Graphical Abstract:**

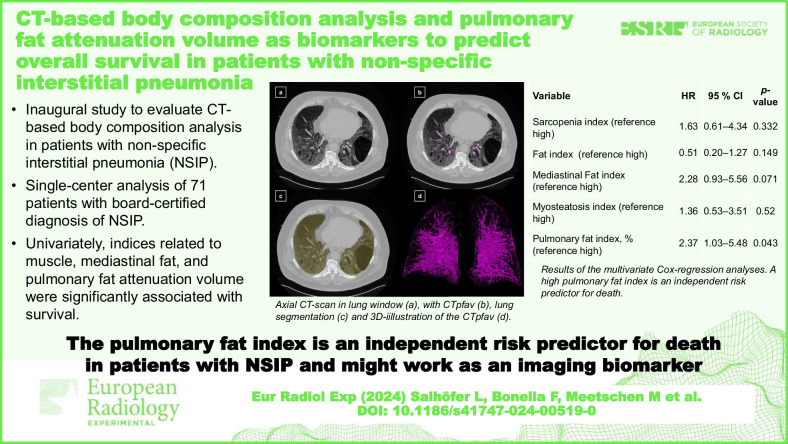

## Background

Non-specific interstitial pneumonia (NSIP) is the second most frequent form of interstitial lung disease (ILD), mostly secondary to autoimmune disease, infection, or drug consumption. NSIP can be distinguished into fibrotic and nonfibrotic according to the extent of inflammation and fibrosis at high-resolution computed tomography (CT) or biopsy [1]. The prognosis can highly vary, as some patients may maintain a stable status or improve under immunosuppressive treatment, but progression to end-stage fibrosis can also occur [1–4]. The gender-age-physiology index has been validated to predict mortality at 1 year, but biomarkers to predict long-term survival are still needed [5, 6].

Factors related to body composition (*e.g*., sarcopenia) are associated with an altered course of chronic diseases, including overall survival (OS) [7, 8]. So far, the body mass index (kg/m^2^) is the most widely used parameter to assess body composition, neglecting its significant limitations as it is influenced by ethnicity, sex, and tissue composition [9–11]. While there are several studies on the influence of body composition on OS in ILD, employing manual or semiautomatic segmentations [12–15], specific studies in NSIP are lacking.

In 2006, Lee et al showed no differences in the volume of anterior mediastinal fat but unveiled a characteristic, convex shape in patients with diagnosed NSIP without deducting any influence on the disease course [16]. O’Callaghan et al have recently established a correlation between the quotient of CT pulmonary fat attenuation volume (CTpfav) by total lung volume and lung function parameters and radiologic features in a cohort of patients with idiopathic pulmonary fibrosis (IPF) employing HU-thresholding (–40 to –200 HU) on semiautomatic lung segmentations [17]. This provides more evidence for the long-established relationship between pulmonary fat metabolism and fibrotic lung processes. Although the evidence for the link between lipid metabolism and fibrosis in NSIP remains to be investigated, multiple studies link dysregulated lipid metabolism to various pulmonary diseases [18, 19]. In patients with IPF, increased pulmonary lipid deposition was observed in human patients and fibrosis mice models proceeding to the maximal extent of fibrosis [20, 21]. As progressive fibrosis is associated with poorer survival, pulmonary fat assessment might be an intriguing prognostic factor in ILD.

Machine learning advances have led to the creation of innovative methods for body composition analysis (BCA) feature extraction with fully automatic segmentation on CT scans and allowed the investigation of more specific biomarkers including the volume of body components (*e.g*., subcutaneous adipose tissue) and organs (*e.g*., lung) [22–25] or the characteristics of individual voxels for tissue characterization (*e.g*., HU-thresholding).

The presented study aimed to introduce fully automatic CTpfav segmentation using the latest machine learning-based developments. Furthermore, our goal was to identify imaging biomarkers as potential predictors of OS in patients with NSIP.

## Methods

### Cohort definition criteria

Initially, all patients diagnosed with NSIP according to an interdisciplinary board decision at our institution and at least one clinical follow-up were considered for inclusion. Missing imaging data (needed chest CT with ≤ 1.5-mm slice thickness reconstructed with a soft-tissue kernel), lung transplantation, and absent lung function testing within 90 days of CT were exclusion criteria.

### Body compositions analysis

A pretrained network (“Body and Organ Analysis”) using a nnU-Net architecture variant that enables highly accurate automatic tissue segmentation in CT scans and various organ segmentations was used to extract the body composition data [22, 23]. All CT data were obtained on a 64-slice scanner (SOMATOM Definition AS, Siemens Healthineers, Erlangen, Germany) with an automatic dose modulation at 120 kV, 0.55 pitch factor, slice thickness of 1.0 or 1.5 mm, 1.0 mm increment and soft-tissue convolutional kernel. The used variant of the network normalizes all CT images to a specific slice thickness (5 mm for BCA, 1.5 mm for organ volumetry) and extracts body composition features within the thoracic cavity excluding the limbs with high segmentation accuracy [23]. The employed BCA model was trained on 300 and validated on 60 separate CT scans (including whole-body, thoracic, abdominal, and neck imaging) with 5-fold cross-validation and incorporates a combination of modern segmentation mechanisms and HU-thresholding for specific tissue types (*e.g*., intra- and inter-muscular adipose tissue is thresholded between –190 and –30 HU within the muscle segmentation) [23].

The following raw parameters were extracted as volumes (mL): bone, muscle, subcutaneous adipose tissue, visceral adipose tissue, intra- and inter-muscular adipose tissue, epicardial adipose tissue, and mediastinal adipose tissue. The investigated parameters in the study were defined by combining selected raw BCA features with the bone volume (Sarcopenia index, Fat index, and Mediastinal Fat index) and total adipose tissue volume (Myosteatosis index) for normalization. Furthermore, the pulmonary fat attenuation volume (CT_pfav_) was assessed by HU-thresholding all fat isodense voxels (HU –200 to –60) within the lung segmentations [17, 23] without any further post-processing. Dividing the CTpfav by the total pulmonary volume yielded the Pulmonary fat index (PFI).

$${Sarcopenia\; index}=\frac{{Muscle} \, [{mL}]}{{Bone} \, [{mL}]}$$

$${Myosteatosis\; index} \, \left[ \% \right]=\frac{{IMAT} \, [{mL}]}{{TAT} \, [{mL}]}{\times}100$$

$${Fat\; index}=\frac{{TAT} \, [{mL}]}{{Bone} \, [{mL}]}$$

$${Mediastinal\; Fat\; index}=\frac{{MAT} \, [{mL}]}{{Bone} \, [{mL}]}$$

$${Pulmonary\; Fat\; index} \, [ \% ]=\frac{{CTpfav} \, [{mL}]}{{Lung\; volume} \, [{mL}]}x100$$

(Figures 1, 2)

**Fig. 1:**
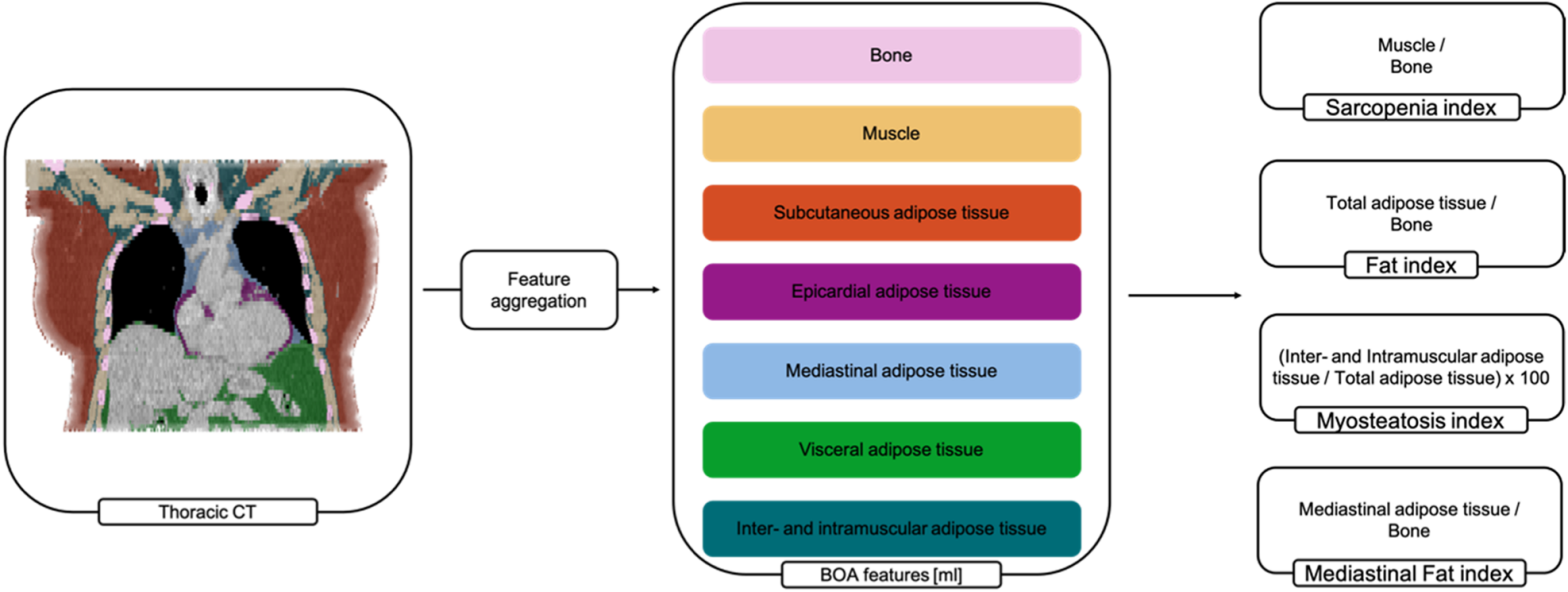
Visualization of the body composition analysis feature extraction and index aggregation. The Body and Organ analysis (BOA) network detects the different features within the thoracic computed tomography scan with the exclusion of the limbs. A combination of those raw features is defined as the Sarcopenia, Myosteatosis, Fat, Mediastinal fat, and Pulmonary fat indices. For better comprehensibility, the tissues were coded in color as follows: pink, bone; yellow, muscle; orange, subcutaneous adipose tissue; green, visceral adipose tissue; light blue, mediastinal adipose tissue; purple, epicardial adipose tissue

**Fig. 2:**
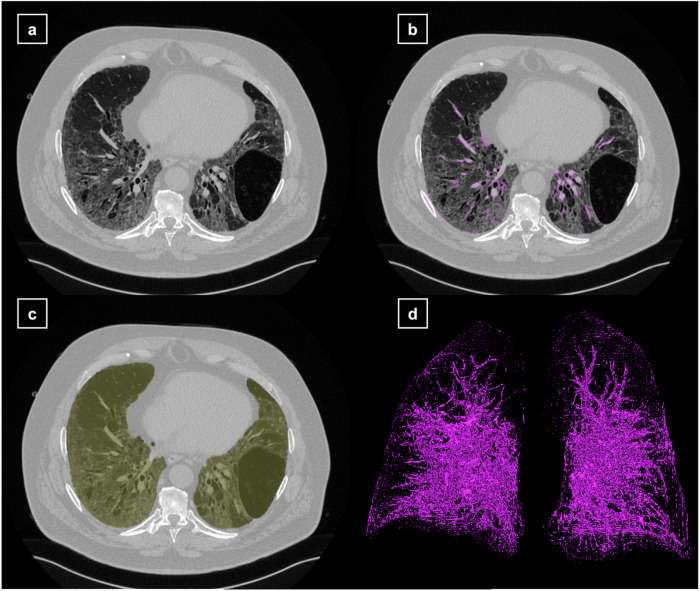
Visualization of the CTpfav and lung segmentation. Illustration of the fully automated lung segmentation and the CTpfav coded in colors: light yellow, lung; pink, CTpfav. **a** Axial CT slice. **b** Axial CT slice combined with the CTpfav segmentation. **c** Axial CT slice combined with the lung segmentation. **d** Three-dimensional illustration of the CTpfav. CT, Computed tomography; CTpfav, CT Pulmonary fat attenuation volume

### Clinical definitions

Sarcopenia is characterized by a loss of muscle mass, strength, or quality, which is related to an increased risk of death in elderly patients [26, 27]. The degree of muscular fatty infiltration (myosteatosis) can be used as a parameter for skeletal muscle quality and strength apart from mass or volume to measure fatty degeneration [28, 29]. For this purpose, the Myosteatosis index was created by using intra- and inter-muscular adipose tissue as a part of total adipose tissue in this study. Besides sarcopenia, cachexia in fibrotic lung/end-stage lung disease can be associated with fat loss [30]. Therefore, several indices with relation to fat were included in the study using mediastinal adipose tissue and total adipose tissue volume normalized to the bone volume.

### Statistical analysis

#### Evaluation of BCA index expression

The cohort was separated into two subgroups by survival with a cutoff at 5 years after the CT scan for the statistical analysis examining the impact of the clinical outcome on the BCA indices (survival < 5 years = 32 patients; survival > 5 years = 39 patients). Normal distribution was confirmed by a D’Agostino and Pearson test. For normally distributed data, a two-tailed *t*-test assessed statistical significance; otherwise Mann–Whitney *U* test was applied.

#### Univariate survival analysis

The cohort was separated into two groups at the median (< median = low; ≥ median = high) for each index to examine the impact of the extracted BCA indices on the OS. The OS of those groups was analyzed by using the Kaplan–Meier method and a log-rank test. Patient characteristics were compared using a two-tailed *t*-test for continuous and a Fisher’s exact test for categorical variables, respectively. Patient survival was assessed by data retrieval of death registers.

#### Multivariate Cox-regression for risk of death

An adjusted multivariate Cox regression analysis was performed to determine whether the BCA indices are independent risk predictors. All indices were investigated as categorical variables using their specific median as a cutoff value. As the BCA indices are linked by distinct body volumes, each of the BCA indices was individually analyzed together with the available clinical and demographic parameters by entering all variables in a single step (enter-method). The value for the diffusion capacity of the lung for carbon monoxide (DLCO) was excluded from this analysis since only 51 patients were able to perform the test. The analyses were performed in Python using the PyCharm software and the lifelines package (0.27.7).

If not otherwise specified, *p*-values lower than 0.05 were considered significant, and the analyses were conducted and displayed with GraphPad Prism (10.1.0) for MacOS.

## Results

### Study population

**Fig. 3:**
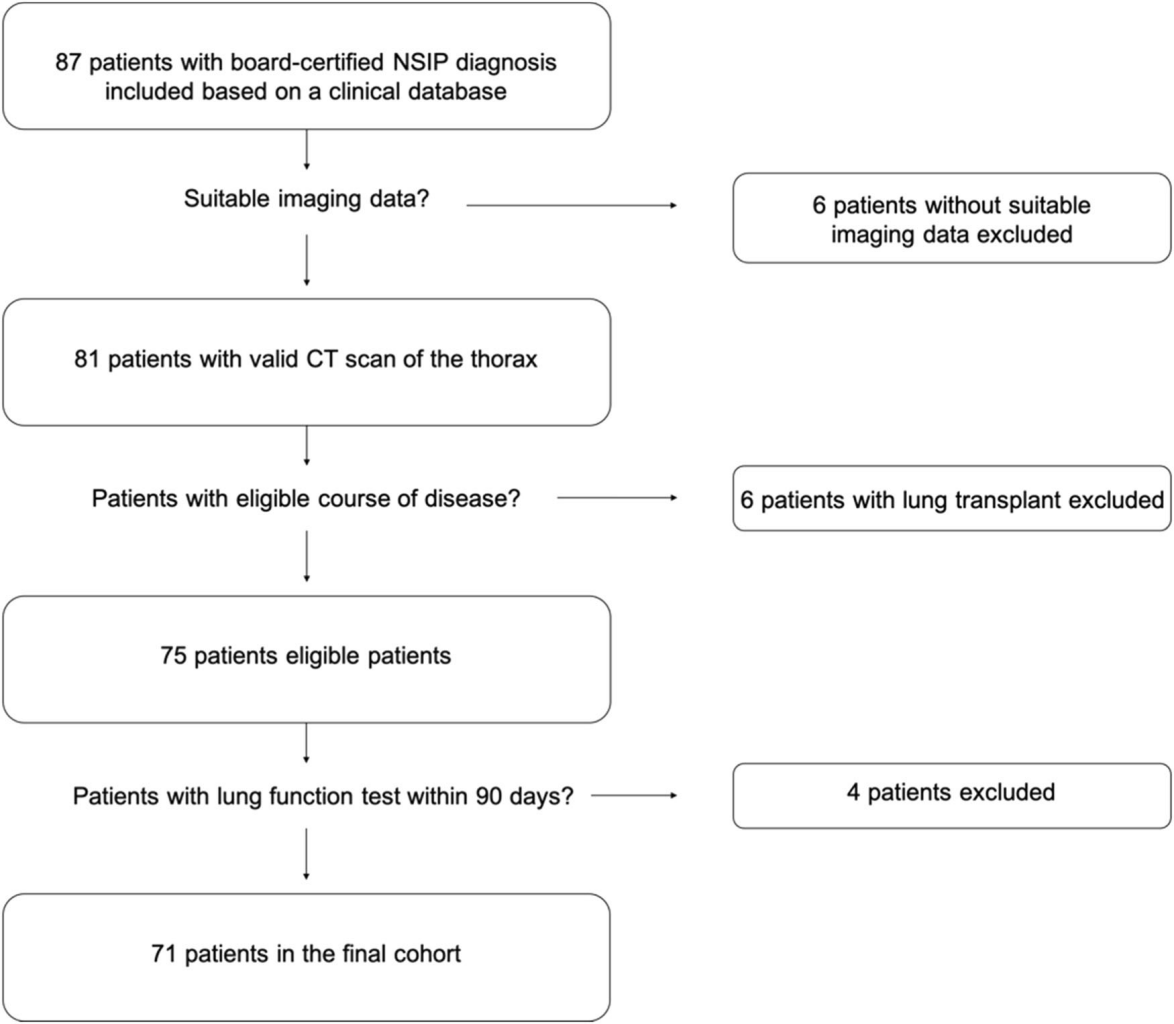
Flow chart of the cohort extraction process. Initially, 87 patients were included in the base cohort. After filtering out patients without a thoracic computed tomography scan, with lung transplantation, and missing lung function data, 71 patients remained as the final cohort

Of 87 patients diagnosed with NSIP by an interdisciplinary ILD board at our institution and at least one clinical follow-up, six were excluded because of unsuitable imaging data. Additionally, six patients who received a lung transplant and four patients with missing lung function tests within 90 days of the CT scan were excluded. Thus, the final cohort comprised 71 individuals (39 females, 32 males), with a median age of 65 years (IQR 53–74.5) at the time of the CT scan. Of these 71 patients, 38 (54%) deceased by March 2023. The original diagnosis was made between December 1998 and February 2015, and the CT scan was performed between August 2009 and February 2018. Sixty-one patients were classified as fibrotic NSIP based on CT morphologic changes decided upon consensus by two experienced thoracic radiologists (7 and 9 years of experience) in a joint image review (Table 1, Fig. 3).

**Table 1:** Demographics and clinical characteristics of the studied patients

Variable	Investigated cohort (*n* = 71)
Age, years^a^	65 (21.5)
Sex, female	39 (55%)
Body mass index, kg/m^2a^	29 (11)
Smoking history, yes (current and ex)	34 (48%)
Interval from diagnosis to CT examination, months^a^	17 (58)
Gender-age-physiology index^a^	4 (2.5)
Fibrotic NSIP	61 (86%)
Pulmonary function test	
Forced vital capacity, %^a^	62 (23.5)
DLCO, mL/mmHg/min^a^ (*n* = 51)	41 (19.5)
Comorbidities	
Cardiovascular comorbidities, yes	48 (68%)
Diabetes mellitus type 2, yes	22 (31%)

*DLCO* Diffusion capacity of the lung for carbon monoxide, *NSIP* Non-specific interstitial pneumonia

^a^ Data are medians with interquartile ranges

### BCA index expression in relation to survival time

**Fig. 4:**
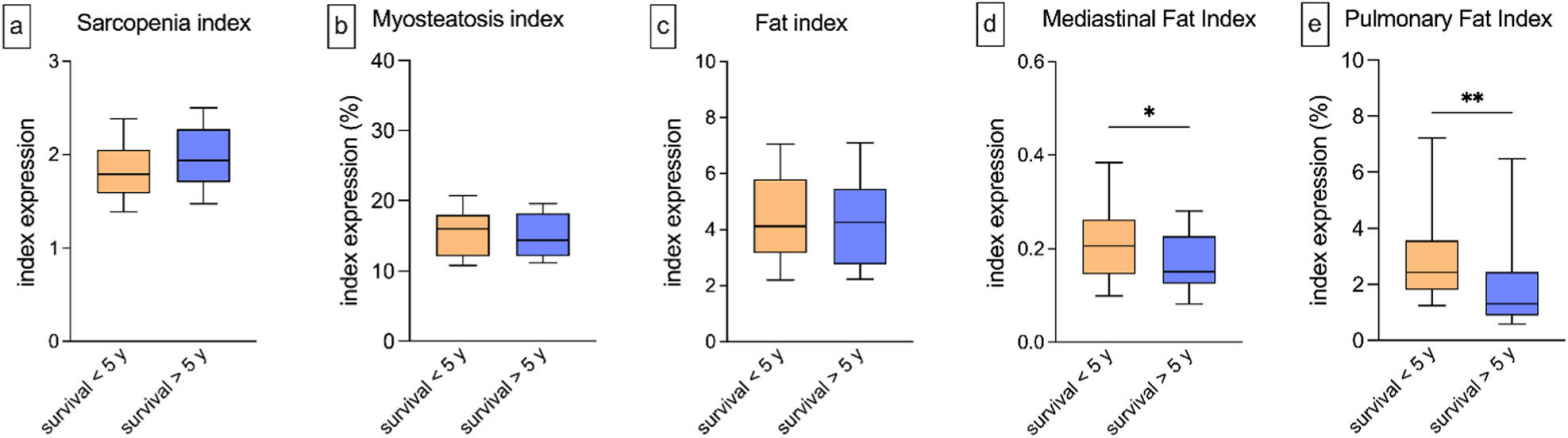
Expression of the BCA indices based on the overall survival of more or less than 5 years. The expression of the BCA-derived imaging indices was studied in two groups: those who survived more or less than 5 years of the CT scan. There were no statistically significant differences between those two groups for the Sarcopenia (**a**, mean for survival < 5 years, 1.82 *versus* > 5 years, 1.97), Fat (**c**, mean for survival < 5 years, 4.47 *versus* > 5 years, 4.40) and Myosteatosis index (**b**, mean for survival < 5 years, 15.55 *versus* > 5 years, 15.26). Patients with a survival of less than 5 years had significantly higher Mediastinal Fat (**d**, mean for survival < 5 years, 0.22 *versus* > 5 years, 0.17, *p* = 0.015) and Pulmonary fat index (**e**, mean for survival < 5 years, 3.12% *versus* > 5 years, 2.19%, *p* = 0.001). The whiskers represent the 10th and 90th percentile. (*p < 0.05; **p < 0.01). BCA, Body composition analysis; CT, Computed tomography

Mediastinal Fat index expression was significantly higher in patients with a survival period of less than 5 years compared to those with a survival of more than 5 years: mean ± standard deviation for survival < 5 years, 0.22 ± 0.10 *versus* survival > 5 years, 0.17 ± 0.07 (*p* = 0.015). The PFI was significantly higher in patients with a survival period of under 5 years as well: 3.12% ± 2.44% *versus* 2.19% ± 2.16%, respectively (*p* = 0.001). Analyses of the Sarcopenia, Fat, and Myosteatosis index among patients who had a shorter or longer survival than 5 years after the CT scan revealed no significant differences (*p* ≥ 0.068). Nevertheless, a tendency toward a higher Sarcopenia index in patients with prolonged survival can be observed: 1.97 ± 0.37 *versus* 1.82 ± 0.33, respectively (*p* = 0.068) (Fig. 4).

### Survival analysis for the BCA

**Fig. 5:**
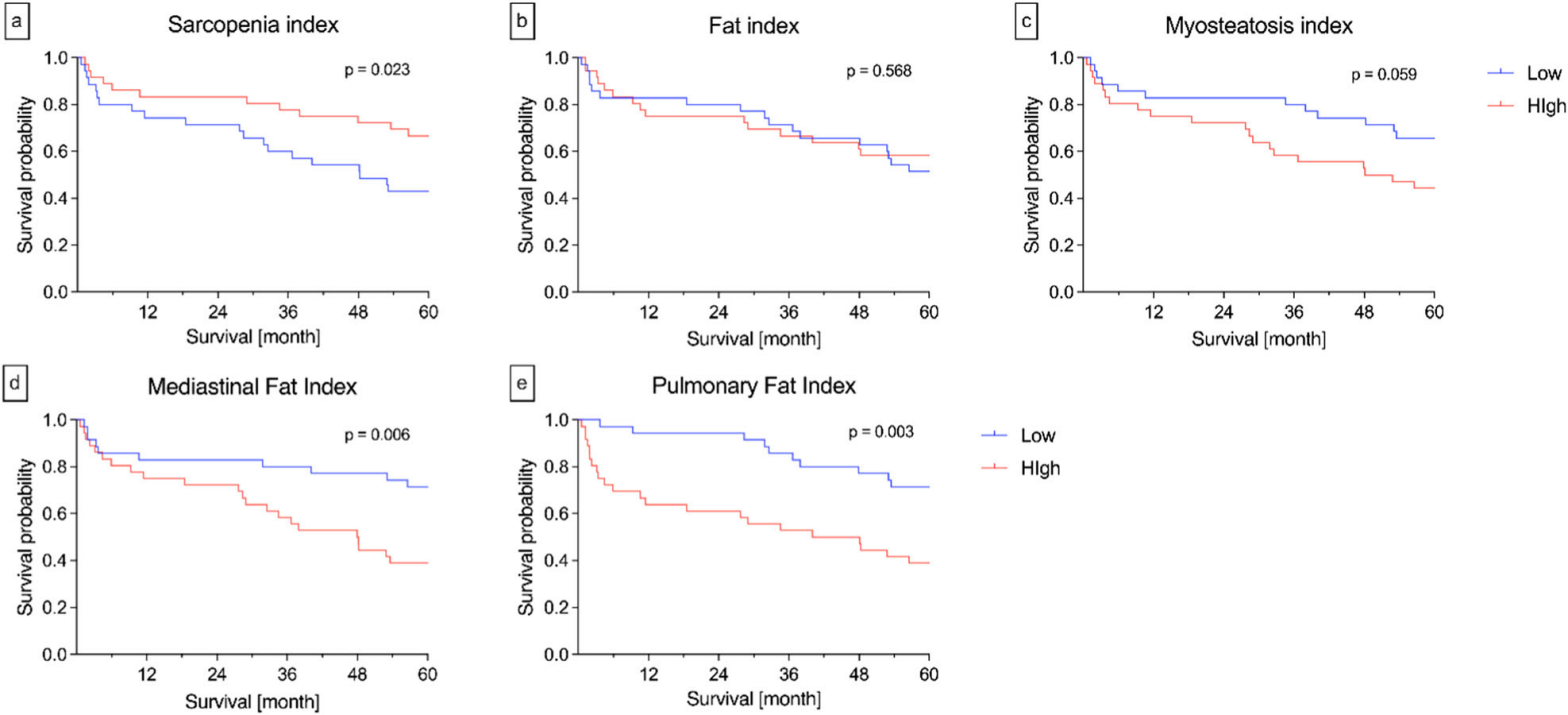
Kaplan–Meier analysis of the OS of all NSIP patients in dependency on the BCA indices. Illustration of the OS of patients with NSIP using the Kaplan–Meier method within a time range of 60 months based on their body composition concerning the BCA indices. The blue curve represents patients with a low expression of the index, and the red curve stands for patients with a high index. Patients with a higher Sarcopenia index demonstrated significantly higher survival probability compared to those with a lower Sarcopenia index (**a**, *p* = 0.023). In contrast, patients with a higher Mediastinal Fat index (**d**, *p* = 0.006) and Pulmonary Fat index (**e**, *p* = 0.003) showed a significantly lower survival probability. No significant differences were observed for the Fat (**b**) and Myosteatosis (**c**) index. BCA, Body composition analysis; NSIP, Non-specific interstitial pneumonia; OS, Overall survival

Patients with a high Sarcopenia index compared to those with low Sarcopenia index had a significantly longer survival time and better survival rates (2-year survival rate, 83% *versus* 71%; 5-year survival rate, 67% *versus* 43%; *p* = 0.023). Regarding the Mediastinal Fat index, we observed a shorter survival time and worse survival rates for patients with a high index expression compared to the group with a low index: 2-year survival rate, 72% *versus* 83%; 5-year survival rate, 39% *versus* 71% (*p* = 0.006). Analysis indicated a reduced survival duration in patients with high PFI in contrast to patients with low PFI coming in hand with worse survival rates: 2-year survival rate, 61% *versus* 94%; 5-year survival rate, 39% *versus* 71% (*p* = 0.003). For the Myosteatosis and Fat-index, no significant results were observed, although a tendency toward a longer median survival time and better survival rates for patients with a low Myosteatosis index can be observed: 2-year survival rate, 83% *versus* 72%, 5-year survival rate, 66% *versus* 44% (*p* = 0.058) (Table 2, Fig. 5).

**Table 2:** Values of body composition analysis indices for all NSIP patients

Indices	Median (IQR)
Sarcopenia index	1.84 (1.63–2.21)
Fat index	4.26 (3.11–5.54)
Myosteatosis index, %	14.96 (12.13–17.96)
Mediastinal fat index	0.17 (0.13–0.24)
Pulmonary fat index, %	2.06 (1.09–3.11)

*IQR* Interquartile range, *NSIP* Non-specific interstitial pneumonia

Since the examined cohort was divided solely based on a BCA index (high *versus* low), it is intriguing to examine the distribution of the additional demographic and clinical parameters (Table 3). For the Sarcopenia, Myosteatosis, and Mediastinal Fat index, there were significant differences in the median age of the patients, *e.g*., low Sarcopenia index, 74 years (IQR 69–77) *versus* high Sarcopenia index, 53 years (IQR 48–62) (*p* < 0.001). The PFI was the only BCA index that exhibited a significant association with survival utilizing the Kaplan–Meier method without any notable variations in age of the two groups: low PFI, 62 years (52–75) *versus* high PFI, 65 years (53–73), *p* = 0.874. Still, patients with high PFI had a significantly lower forced vital capacity (FVC) compared to patients with low PFI: 49.5% (42–64) *versus* low: 69% (62–78), *p* < 0.001.

**Table 3:** Demographic and clinical parameters among the investigated groups of NSIP patients

	Sarcopenia index			Fat index		
	Low	High	*p*-value	Low	High	*p*-value
Age, years^a^	74 (69–77)	53 (48–62)	< 0.001	69.5 (52–75)	63.5 (55–74)	0.985
Sex, male	15	17	0.813	23	9	< 0.001
Body mass index, kg/m^2^	27 (25.5–32)	32 (27–35)	0.066	26.5 (24–29.25)	33 (29–36.5)	< 0.001
Smoking history, yes	13	21	0.098	22	12	0.018
Gender-age-physiology index	5 (4–6)	3.5 (2–5)	0.048	4 (3–5)	5 (2–6)	0.049
Fibrotic NSIP	31	30	0.735	32	29	0.307
Pulmonary function test						
Forced vital capacity, %^a^	65 (49–73.5)	60.5 (53–71)	0.419	65 (53–73.5)	59.5 (48–68)	0.076
DLCO, mL/mmHg/min^a^	40 (31.25–56)	42 (36–50.5)	0.601	42 (35–54)	39 (32–51)	0.664
Comorbidities						
Cardiovascular comorbidities, yes	28	20	0.042	25	23	0.614
Diabetes mellitus type 2, yes	12	10	0.614	5	17	0.004

	Myosteatosis index			Mediastinal fat index		
	Low	High	*p*-value	Low	High	*p*-value
Age, years^a^	58 (50–69.5)	71.5 (60–76)	0.009	57 (47–69)	71.5 (60–76)	< 0.001
Sex, male	7	25	< 0.001	12	20	0.096
Body mass index, kg/m^2^	32 (27–35)	28 (25–33)	0.262	27 (24–31)	32 (28–37.5)	0.003
Smoking history, yes	14	20	0.238	19	15	0.346
Gender-age-physiology index	4 (2–5)	4 (3–6)	0.169	4 (3)	5 (2)	0.633
Fibrotic NSIP	30	31	1.000	32	29	0.307
Pulmonary function test						
Forced vital capacity, %^a^	58 (48–72)	65 (50–74)	0.314	62 (49–72.5)	61.5 (49–72)	0.708
DLCO, mL/mmHg/min^a^	41 (36–48.5)	40 (30–55)	0.993	46 (35–56)	36.5 (31.75–49)	0.220
Comorbidities						
Cardiovascular comorbidities, yes	21	27	0.211	19	29	0.023
Diabetes mellitus type 2, yes	9	13	0.443	5	17	0.004

	Pulmonary fat index		
	Low	High	*p*-value
Age, years^a^	62 (52–75)	65 (53–73)	0.874
Sex, male	18	14	0.813
Body mass index, kg/m^2^	27 (25–35)	30.5 (27–34.25)	0.680
Smoking history, yes	20	14	1.000
Gender-age-physiology index	4 (3–5)	5 (4–6)	0.134
Fibrotic NSIP	28	33	0.189
Pulmonary function test			
Forced vital capacity, %^a^	69 (62–78)	49.5 (42–64)	< 0.001
DLCO, mL/mmHg/min^a^	42 (33–51)	36.5 (33–51.24)	0.558
Comorbidities			
Cardiovascular comorbidities, yes	24	24	0.803
Diabetes mellitus type 2, yes	9	13	0.200

*DLCO* Diffusion capacity of the lung for carbon monoxide, *NSIP* Non-specific interstitial pneumonia

^a^ Data are medians with interquartile ranges

### Multivariate analysis

In multivariate Cox-regression analysis, a high PFI was associated with a significantly increased risk of death compared to patients with a low PFI: hazard ratio (HR) 2.37, 95% confidence interval (CI) 1.03–5.48, *p* = 0.043. Patients with a high Mediastinal Fat index demonstrate a similar HR (2.28, 95% CI 0.93–5.56), but the results did not reach statistical significance (*p* = 0.071). Among the clinical and demographic covariates considered, only age emerged as an independent predictor of mortality in all conducted analyses with only a slight increase in the HR (*e.g*., in the Cox-Regression for the PFI: HR 1.05, 95% CI 1.00–1.10, *p* = 0.043). All other BCA indices and covariates did not show significant results in any analysis (*p* ≥ 0.149) (Table 4, Appendix 1–5).

**Table 4:** Overview of the multivariate Cox-regression analyses results for body composition analysis indices as categorical variables

Variable	HR	95% CI	*p*-value
Sarcopenia index (reference high)	1.63	0.61–4.34	0.332
Fat index (reference high)	0.51	0.20–1.27	0.149
Mediastinal fat index (reference high)	2.28	0.93–5.56	0.071
Myosteatosis index (reference high)	1.36	0.53–3.51	0.52
Pulmonary fat index, % (reference high)	2.37	1.03–5.48	0.043

*HR* Hazard ratio, *ref*. reference, *95% CI* 95% confidence interval

## Discussion

In the present study, we found that the fully automated PFI, derived from thoracic CT scans, seems to be an independent risk predictor for OS in patients with NSIP. Unlike in other chronic diseases, sarcopenia, measured as the Sarcopenia index, demonstrates no substantial association with the OS for this condition.

The main finding of our study is in line with the observations on the role of lipids and their deposition in ILDs, particularly IPF [20, 21]. We were able to establish a correlation between the remaining life span and the PFI, defined as CTpfav/Lung Volume ratio in patients with NSIP. Additionally, the PFI was an independent risk predictor of death (HR 2.37, *p* = 0.043). Our investigations build upon the recent groundwork laid by O’Callaghan et al [17], who were the first to assess pulmonary lipid volume on chest CT scans using HU-thresholding in lung segmentations. They successfully established a correlation among CTpfav, pulmonary function, and radiological characteristics in patients diagnosed with IPF. Furthermore, they demonstrated that patients with IPF exhibited histologically more adipose tissue compared to the control group, along with higher CTpfav values [17]. Since Husseini et al [31] demonstrated that pronounced intraparenchymal pulmonary fat can also be found in fibrotic NSIP histologically, the CTpfav could therefore be relevant for these patients as well. To date, there is no available data on the association between CTpfav and OS in general, especially not for NSIP. However, it is recognized that lipid metabolism influences fibrosis in ILDs, and progressive fibrosis is typically linked with poorer survival outcomes [20, 31, 32]. Therefore, a correlation between a detection method of pulmonary fat and OS could potentially represent a novel imaging biomarker. Due to the division of the cohort at the median PFI, patients with a high PFI had a significantly lower FVC compared to patients with a low index. Since Zappala et al demonstrated that reduced FVC can be associated with poorer survival in fibrotic NSIP, an influence on the results of the univariate OS analysis is possible [33]. Interestingly, we were able to identify the PFI as an independent risk factor in the multivariate analysis and therefore present a promising step forward as we pioneer an association of PFI and OS. Particularly, since pulmonary fat accumulations precede the maximum extent of fibrosis [20], PFI might be an interesting target for future biomarker research.

In healthy elderly people and several chronic conditions, a reduction of muscle volume and increased muscular fatty infiltration is associated with lower survival [26–29]. Additionally, reduced fat volume is associated with poorer prognosis in several diseases [34]. Although differences in body composition have been observed in patients with IPF and at times deducted an association with OS [12–14, 35], there are only sporadic investigations incorporating body composition changes in patients with NSIP, but none correlating it with OS. Guler et al demonstrated a correlation between the severity of ILDs and body composition parameters in a heterogeneous cohort of ILD patients [36]. They assessed body composition with Dual-energy X-ray absorptiometry and focussed on the skeletal muscle index, which correlated with ILD severity measured by DLCO [36]. Although a strong correlation between CT-based BCA and dual-energy x-ray absorptiometry measurements has been demonstrated previously [25], we were unable to replicate the findings reported by Guler et al [36] concerning survival. The Kaplan–Meier analysis of patients with a Sarcopenia index below and a Myosteatosis index above the median of the overall cohort showed a poorer overall survival. Nevertheless, this finding is probably related to the age difference between the two groups, as neither the Sarcopenia nor the Fat or Myosteatosis markers were independent predictor variables in the multivariate approach. These inconsistent findings may arise from the fact that our cohort exclusively comprised NSIP patients, whereas Guler et al studied a heterogeneous group of ILD patients, with IPF being the predominant subtype and NSIP representing only a minority. Our results suggest that cachexia-related BCA features do not have a substantial association with OS in patients with NSIP.

In our analysis, patients with a life expectancy shorter than 5 years after the CT exhibited a significantly higher mediastinal fat volume relative to individual bone volume. A previous study by Lee et al in 2006 compared single-plane CT examinations from patients with a UIP pattern and those with NSIP high-resolution CT patterns [16]. The authors were unable to identify differences in the amount of anterior mediastinal fat. Advancements in imaging and machine learning methodologies have facilitated a more precise assessment of mediastinal fat volume [22, 23]. This more in-depth approach showed group of patients with a higher Mediastinal Fat index had a significantly poorer survival in the Kaplan–Meier analysis in our study. However, the composition of the groups formed by the median split also turned out to be different, particularly in terms of age, and the Mediastinal Fat index could not be elaborated as an independent risk factor. Nevertheless, because of the high hazard ratio and the nearly significant result (HR 2.28, *p* = 0.071), the findings of our study are still intriguing and suggest further investigation of mediastinal fat in future analyses.

This study has several limitations. First, the monocentric study design with a limited cohort size. Subsequent and multicentric studies are warranted to validate the results. Additionally, the cohort size did not allow us to conduct subgroup analyses, for example, to compare survival in fibrotic *versus* non-fibrotic NSIP. Second, we did not include the treatment, which can be very heterogeneous in NSIP, leading to a partial validity of our conclusions in terms of survival predictors. Most importantly, the fully automated CTpfav detection has a potential bias because of partial volume averaging. As the detected density values (–200 to –40 HU) stand between the values of lung and fibrotic changes, increased fibrosis might result in higher CTpfav because of the wider interface. Thus, presumable only CT data with a slice thickness of ≤ 1.5 mm and low increment should be used for this assessment, as lower slice thickness leads to less partial volume averaging. However, further studies are needed to evaluate the impact of CT scanner generation and scan parameters on CTpfav quantification.

To the best of our knowledge, the present study is the first that quantifies pulmonary fat isodense volume in CTs (CTpfav) fully automatically and demonstrates an association between survival and the calculated PFI. From the clinical point of view, more pronounced fatty infiltration of lung tissue seems to be a significant risk factor for poor survival in NSIP, independent of clinical and demographic features (*e.g*., age). Further investigations on mediastinal adipose tissue and survival in ILD are warranted.

## Supplementary information

**Additional file 1:** **Appendix 1.** Multivariate Cox-regression analysis for the Sarcopenia index as a categorical variable of NSIP patients. **Appendix 2.** Multivariate Cox-regression analysis for the Fat index as a categorical variable of NSIP patients. **Appendix 3.** Multivariate Cox-regression analysis for the Mediastinal Fat index as a categorical variable of NSIP patients. **Appendix 4:** Multivariate Cox-regression analysis for the Myosteatosis index as a categorical variable of NSIP patients. **Appendix 5:** Multivariate Cox-regression analysis for the Pulmonary Fat index as a categorical variable of NSIP patients.

## Data Availability

The datasets used and/or analyzed during the current study are available from the corresponding author upon reasonable request.

## Abbreviations

BCA: Body composition analysis
CI: Confidence interval
CT: Computed tomography
CTpfav: CT pulmonary fat attenuation volume
DLCO: Diffusion capacity of the lung for carbon monoxide
FVC: Forced vital capacity
HR: Hazard ratio
ILD: Interstitial lung disease
IPF: Idiopathic pulmonary fibrosis
IQR: Interquartile range
NSIP: Non-specific interstitial pneumonia
OS: Overall survival
PFI: Pulmonary fat index
