# Supplementary material for: CT-based body composition analysis and pulmonary fat attenuation volume as biomarkers to predict overall survival in patients with non-specific interstitial pneumonia
Source: Eur Radiol Exp. 2024 Oct 14;8:114. doi: 10.1186/s41747-024-00519-0 (PMC11473462; doi:10.1186/s41747-024-00519-0)
Supplement: Supplementary file 1 — Additional file 1: Appendix 1. Multivariate Cox-regression analysis for the Sarcopenia index as a categorical variable of NSIP patients. Appendix 2. Multivariate Cox-regression analysis for the Fat index as a categorical variable of NSIP patients. Appendix 3. Multivariate Cox-regression analysis for the Mediastinal Fat index as a categorical variable of NSIP patients. Appendix 4: Multivariate Cox-regression analysis for the Myosteatosis index as a categorical variable of NSIP patients. Appendix 5: Multivariate Cox-regression analysis for the Pulmonary Fat index as a categorical variable of NSIP patients. [file 41747_2024_519_MOESM1_ESM.pdf]

CT-based body composition analysis and pulmonary fat attenuation volume as biomarkers to predict overall survival in patients with non-specific interstitial pneumonia

ELECTRONIC SUPPLEMENTARY MATERIAL

Appendix 1. Multivariate Cox-regression analysis for the Sarcopenia index as a categorical variable of NSIP patients

Variable	HR	95 % CI	*p*-value
Age, years (continuous)	1.07	1.02–1.13	0.007
Sex, (reference male)	1.20	0.53–2.74	0.660
Body mass index, kg/m2 (continuous)	0.97	0.90–1.04	0.422
Smoking history, (reference yes)	1.67	0.71–3.89	0.238
Gender-age-physiology index	1.25	0.90–1.75	0.182
**Pulmonary Function Test**			
Forced vital capacity, l (continuous)	0.98	0.95–1.02	0.319
DLCO, mL/mmHg/min (continuous)	/	/	/
**Comorbidities**			
Cardiovascular comorbidities, (reference	0.75	0.29–1.92	0.774
Diabetes mellitus type 2, (reference yes)	0.89	0.42–1.92	0.774
**BCA index**			
Sarcopenia index (reference high)	1.63	0.61–4.34	0.332

BCA body composition analysis, *DLCO* diffusion capacity of the lung for carbon monoxide, *HR* hazard ratio, *kg* kilogram, *m^2* meters squared, *mo* month, *l* liter, *mL* milliliter, *min* minute, *mmHg* millimeters of mercury, *NSIP* Non-specific interstitial pneumonia, *95 % CI* 95 % confidence interval

Appendix 2. Multivariate Cox-regression analysis for the Fat index as a categorical variable of NSIP patients

**Variable**	**HR**	**95 % CI**	***p*-value**
Age, years (continuous)	1.07	1.02–1.12	0.006
Sex, (reference male)	1.00	0.43–2.35	0.993
Body mass index, kg/m2 (continuous)	1.01	0.94–1.09	0.777
Smoking history, (reference yes)	1.79	0.76–4.22	0.181
Gender-age-physiology index	1.19	0.87–1.64	0.278
**Pulmonary Function Test**			
Forced vital capacity, l (continuous)	0.98	0.94–1.01	0.181
DLCO, mL/mmHg/min (continuous)	/	/	/
**Comorbidities**			
Cardiovascular comorbidities, (reference	0.63	0.24–1.63	0.336
Diabetes mellitus type 2, (reference yes)	1.09	0.48–2.44	0.84
**BCA index**			
Fat index (reference high)	0.51	0.20–1.27	0.149

BCA body composition analysis, *DLCO* diffusion capacity of the lung for carbon monoxide, *HR* hazard ratio, *kg* kilogram, m^2 meters squared, *mo* month, *l* liter, *mL* milliliter, *min* minute, *mmHg* millimeters of mercury, *NSIP* Non-specific interstitial pneumonia, *95 % CI* 95 % confidence interval

Appendix 3. Multivariate Cox-regression analysis for the Mediastinal Fat index as a categorical variable of NSIP patients

**Variable**	**HR**	**95 % CI**	***p*-value**
Age, years (continuous)	1.05	1.00–1.10	0.045
Sex, (reference male)	0.96	0.40–2.31	0.929
Body mass index, kg/m2 (continuous)	0.96	0.89–1.03	0.241
Smoking history, (reference yes)	2.13	0.89–5.11	0.09
Gender-age-physiology index	1.24	0.89–1.73	0.199
**Pulmonary Function Test**			
Forced vital capacity, l (continuous)	0.98	0.95–1.02	0.281
DLCO, mL/mmHg/min (continuous)	/	/	/
**Comorbidities**			
Cardiovascular comorbidities, (reference	0.76	0.29–1.98	0.579
Diabetes mellitus type 2, (reference yes)	0.68	0.31–1.51	0.342
**BCA index**			
Mediastinal fat index (reference high)	2.28	0.93–5.56	0.071

BCA body composition analysis, *DLCO* diffusion capacity of the lung for carbon monoxide, *HR* hazard ratio, *kg* kilogram, m^2 meters squared, *mo* month, *l* liter, *mL* milliliter, *min* minute, *mmHg* millimeters of mercury, *NSIP* Non-specific interstitial pneumonia, *95 % CI* 95 % confidence interval

Appendix 4: Multivariate Cox-regression analysis for the Myosteatosis index as a categorical variable of NSIP patients

**Variable**	**HR**	**95 % CI**	***p*-value**
Age, years (continuous)	1.06	1.01–1.11	0.015
Sex, (reference male)	1.00	0.35–2.91	0.996
Body mass index, kg/m2 (continuous)	0.98	0.92–1.05	0.611
Smoking history, (reference yes)	1.72	0.75–3.95	0.200
Gender-age-physiology index	1.20	0.88–1.65	0.254
**Pulmonary Function Test**			
Forced vital capacity, l (continuous)	0.98	0.95–1.01	0.266
DLCO, mL/mmHg/min (continuous)	/	/	/
**Comorbidities**			
Cardiovascular comorbidities, (reference	0.76	0.29–1.98	0.574
Diabetes mellitus type 2, (reference yes)	0.86	0.40–1.83	0.693
**BCA index**			
Myosteatosis index (reference high)	1.36	0.53–3.51	0.52

BCA body composition analysis, *DLCO* diffusion capacity of the lung for carbon monoxide, *HR* hazard ratio, *kg* kilogram, m^2 meters squared, *mo* month, *l* liter, *mL* milliliter, *min* minute, *mmHg* millimeters of mercury, *NSIP* Non-specific interstitial pneumonia, *95 % CI* 95 % confidence interval

Appendix 5: Multivariate Cox-regression analysis for the Pulmonary Fat index as a categorical variable of NSIP patients

**Variable**	**HR**	**95 % CI**	***p*-value**
Age, years (continuous)	1.05	1.00–1.10	0.034
Sex, (reference male)	1.45	0.62–3.35	0.391
Body mass index, kg/m2 (continuous)	0.98	0.92–1.05	0.552
Smoking history, (reference yes)	1.71	0.75–3.93	0.203
Gender-age-physiology index	1.25	0.91–1.72	0.173
**Pulmonary Function Test**			
Forced vital capacity, l (continuous)	1.00	0.96–1.04	0.927
DLCO, mL/mmHg/min (continuous)	/	/	/
**Comorbidities**			
Cardiovascular comorbidities, (reference	0.77	0.30–1.99	0.591
Diabetes mellitus type 2, (reference yes)	0.87	0.40–1.91	0.729
**BCA index**			
Pulmonary fat index, % (reference high)	2.37	1.03–5.48	0.043

BCA body composition analysis, *DLCO* diffusion capacity of the lung for carbon monoxide, *HR* hazard ratio, *kg* kilogram, m^2 meters squared, *mo* month, *l* liter, *mL* milliliter, *min* minute, *mmHg* millimeters of mercury, *NSIP* Non-specific interstitial pneumonia, *95 % CI* 95 % confidence interval
